# Terapia fibrinolítica sistêmica no tromboembolismo pulmonar

**DOI:** 10.1590/1677-5449.007316

**Published:** 2017

**Authors:** José Manuel Ceresetto, Marcos Arêas Marques

**Affiliations:** 1 Hospital Britânico de Buenos Aires, Buenos Aires, Argentina.; 2 Universidade do Estado do Rio de Janeiro – UERJ, Hospital Universitário Pedro Ernesto – HUPE, Rio de Janeiro, RJ, Brasil.

**Keywords:** fibrinolíticos, embolia pulmonar, hemorragia

## Abstract

O tromboembolismo pulmonar permanece como um grande desafio terapêutico para os médicos especialistas, pois, apesar de todo investimento e desenvolvimento em seu diagnóstico, profilaxia e tratamento, essa condição continua sendo a principal causa de morte evitável em ambiente hospitalar. Ainda restam muitas dúvidas em relação a qual perfil de paciente vai se beneficiar de fato da terapia fibrinolítica sistêmica, sem ficar exposto a um grande risco de sangramento. A estratificação de risco e a avaliação do prognóstico do evento, através de escores clínicos de insuficiência ventricular direita, marcadores de dilatação e disfunção do ventrículo direito e avaliação da massa trombótica, associados ou de forma isolada, são ferramentas que podem auxiliar na identificação do paciente que irá se beneficiar dessa terapia. Os únicos consensos em relação à terapia fibrinolítica no tratamento do tromboembolismo pulmonar são: não deve ser indicada de forma rotineira; nenhum dos escores ou marcadores, isoladamente, devem justificar seu uso; e os pacientes com instabilidade hemodinâmica são os mais beneficiados. Além disto, deve-se avaliar cada caso em relação ao risco de sangramento, especialmente no sistema nervoso central.

## INTRODUÇÃO

Os primeiros relatos publicados sobre o uso da trombolisina (mistura de estreptoquinase e plasminogênio) em 43 pacientes (30 normotensos) para o tratamento fibrinolítico do tromboembolismo pulmonar (TEP), baseados em estudos realizados entre 1958 e 1964 no Instituto Sloan Kettering em Nova Iorque, demonstraram resultados que não eram motivo para empolgação, tampouco para consternação^1^. Até os dias atuais, observamos um cenário similar, não havendo melhora significativa nos resultados publicados com o passar do tempo, ao mesmo tempo em que não se observam complicações proibitivas ao seu uso, e ainda permanece a dúvida de quais pacientes teriam maior benefício com essa terapêutica.

Nos últimos 30 anos, a mortalidade do TEP nos primeiros meses permaneceu acima dos dois dígitos (17%)^2^^-^ 4. A morte secundária ao TEP ocorre pela insuficiência aguda do ventrículo direito (VD) causada pela brusca obstrução de uma artéria pulmonar que culmina em um choque cardiogênico pela falha progressiva da pré-carga do ventrículo esquerdo (VE)^5^.

A terapia fibrinolítica sistêmica (TFS) pode, em pouco tempo, diminuir a massa trombótica, corrigir a hipertensão pulmonar (HP) secundária ao TEP, otimizar os parâmetros hemodinâmicos e melhorar a oxigenação e o fluxo capilar, levando ao rápido alívio dos sintomas, além de eliminar os trombos remanescentes da pelve ou de membros inferiores (MMII), reduzindo o risco de recidiva de embolia pulmonar^6^.

O único trabalho que comparou a mortalidade da TFS com a causada pelo uso de heparina no tratamento do TEP maciço com instabilidade hemodinâmica (IH) foi suspenso precocemente devido à elevada taxa de mortalidade no braço da heparina, enquanto que no braço da TFS essa taxa foi de zero^7^. Desde então, os pacientes com TEP e IH são os principais candidatos à TFS. Por outro lado, o risco de morte, a recorrência do TEP e o desenvolvimento da HP crônica são mais frequentes nos pacientes com insuficiência aguda de VD (TEP submaciço)^8^; em vista disso, por muitos anos se discutiu a ampliação do uso da TFS nesses pacientes, por serem muito mais frequentes do que aqueles com IH^9^. Porém, os trabalhos publicados em que se comparou o uso de heparina com a TFS no TEP submaciço não demonstraram redução significativa da mortalidade com o uso da TFS e, portanto, o uso desta se estabeleceu apenas naqueles 3 a 5% dos pacientes de TEP que apresentam IH^8^.

Os defensores da TFS no TEP submaciço argumentam que as populações avaliadas nos ensaios clínicos são enviesadas, como por exemplo no estudo *Management Strategies and Prognosis of Pulmonary Embolism-*3 (MAPPET 3), bi qual somente 31% dos pacientes tinham dilatação do VD, resultando em uma mortalidade no braço da heparina de 2%, bem abaixo dos 10% esperados e que, ao permitir o resgate com a TFS nesse braço, favoreceu a sobrevida do grupo controle^10^. Há também crítica em relação ao “n” atingido, pois, somando todos os pacientes dos trabalhos randomizados, teremos um pouco mais de 1.000 que receberam a TFS, o que seria uma amostra insuficiente^10^. Há que se ressaltar, porém, que esses trabalhos também demostram redução significativa dos parâmetros secundários, como melhora rápida da HP e redução da massa trombótica e da dilatação do VD^11^.

Naturalmente, um dos grandes problemas relacionados ao uso da TFS é o aumento do número de sangramentos maiores (10 a 20%), particularmente no sistema nervoso central (SNC), com uma morbimortalidade de até 55%, o que limita claramente o benefício de seu uso^12^^,^ 13.

Portanto, atualmente os consensos sobre o manejo do TEP são claros e objetivos em afirmar que a única indicação precisa da TFS é o paciente com IH^3^^,^ 14 ^,^ 15. O nono consenso da *American College of Chest Physicians* (ACCP) de 2012 sugere a TFS apenas no TEP grave com falência de VD e sem IH e, mesmo assim, com um nível de evidência muito fraco (2C) e após avaliação individualizada dos riscos e benefícios^14^.

Estudo recém-publicado descreve a experiência do uso de TFS em 20% dos hospitais americanos. Apenas 2,1% dos mais de 90.000 pacientes com diagnóstico de TEP entre os anos de 2008 e 2011 receberam TFS, e no subgrupo de pacientes que necessitaram vasopressores para manutenção da pressão arterial (PA) apenas 13,2% a receberam^16^^,^ 17.

O objetivo deste artigo é discutir os riscos e benefício da TFS no tratamento do TEP e quais pacientes se beneficiariam de fato. Para isso, foi feita uma revisão bibliográfica dos artigos publicados em inglês na PubMed nos últimos 10 anos.

## ESTRATIFICAÇÃO DE RISCO NO TEP

Para identificarmos o paciente ideal para o uso de TFS, foi proposto classificá-los segundo o risco de vida e o prognóstico do evento. Em muitos estudos procuraram-se marcadores secundários de gravidade do TEP, denominados de complicações do TEP, como a necessidade da repetição da TFS, o uso de suporte ventilatório e hemodinâmico com agentes inotrópicos para manutenção da PA, ressuscitação cardiopulmonar e recidiva do evento^18^.

O TEP pode ser classificado como de baixo (normotensão sem falência de VD), moderado (normotensão com falência de VD) e alto risco (hipotensão refratária)^19^^,^ 20.

Essa classificação, apesar de prática, apresenta um problema relativo à definição da falência do VD, seja por dilatação ou disfunção, que se baseia em critérios pouco claros e subjetivos, e isso acaba imputando em uma incapacidade de predizer com precisão a sua morbimortalidade^20^^-^ 22. Esse fato expõe a necessidade de se avaliar marcadores de gravidade mais fidedignos para ajudar na definição da terapêutica. Ou seja, definir se basta a anticoagulação plena com heparina ou se é necessário utilizar a TFS nesse perfil de pacientes que poderiam evoluir para um pior prognóstico^6^.

Certamente, esse é o principal desafio que se enfrenta na terapêutica do TEP.

### Critérios clínicos de prognóstico

Exceto o paciente com PA sistólica menor que 90 mmHg por choque cardiogênico, a avaliação de determinados parâmetros clínicos como congestão hepática, turgência jugular, sopro sistólico em topografia de valva pulmonar e, especialmente, sinais indiretos de choque (hipotermia, confusão mental, síncope, taquicardia, hipotensão progressiva e hipoxemia que requer assistência respiratória), melhoraram a capacidade preditiva de complicações no TEP^23^.

O escore de risco clínico mais reconhecido é o PESI (*Pulmonary Embolism Severity Index*), que classifica os pacientes em cinco categorias de risco. Os pacientes das categorias de menor risco (categorias I e II) tiveram uma mortalidade de 2% ao mês, enquanto que nos de maior risco (categoria V) esta foi de 14%^24^. Em 2010, é publicado o PESI simplificado, com cinco parâmetros de fácil mensuração: idade > 80 anos, câncer ativo, doença cardiopulmonar crônica, frequência cardíaca (FC) > 110 bpm e PA sistólica < 100 mmHg, cada um valendo um ponto Esse escore é validado e útil para identificar os pacientes com bom prognóstico^25^ e separa aqueles em TEP de baixo risco (PESI 0), com mortalidade de 1,1% ao mês, daqueles de moderado/alto risco (PESI ≥ 1), com mortalidade de 8,9% ao mês .

### Dilatação do ventrículo direito

a) Eletrocardiograma: Apesar de ser um método de fácil execução, não aparece na maioria dos algoritmos de avaliação do TEP. Pode apresentar alterações como um novo bloqueio de ramo direito, supra ou infraelevação de segmento ST anteroseptal e/ou padrão S1Q3T3.
b) Ecocardiograma: é uma ferramenta muito útil na avaliação da falência do VD, pois exibe uma imagem dinâmica da sua função contrátil, pode ser executado à beira do leito em um paciente instável e não é invasivo, o que permite a sua repetição regular no acompanhamento da TFS. Além disto, permite mensurar e avaliar o tamanho do VD e a sua relação com o VE (índice VD/VE), a presença de hipocinesia da parede livre do VD, o movimento paradoxal do septo interventricular (sinal de McConell), a perda do colapso inspiratório da veia cava inferior, a intensidade da HP e a regurgitação da valva tricúspide^23^^,^ 26. Possui limitações como ser uma técnica examinador dependente e não ter ainda padronização nos critérios de definição de falência do VD.
c) Tomografia computadorizada (TC): técnica muito útil na avaliação do diâmetro do VD e sua relação com o de VE (na altura das valvas tricúspide e mitral, se maior que 0,9 configura-se um VD dilatado). Com os dados do *Prospective Investigation of Pulmonary Embolism Diagnosis* (PIOPED) II, observou-se que esse parâmetro se associava a uma maior mortalidade (RR: 1,27)^27^, e uma metanálise observou que esse parâmetro aumentava em 2,5 vezes a mortalidade^28^. Seu valor como método único é limitado e tende a magnificar a quantidade de pacientes com o VD dilatado (no PIOPED II, 50% dos pacientes normotensos tinham o VD dilatado).
d) Peptídeo natriurético tipo B (BNP) e seu fragmento terminal NT-pro-BNP: esses biomarcadores secretados pelo miocárdio quando exposto à dilatação excessiva de suas fibras, é um marcador indireto da dilatação do VD. Em uma revisão de 13 estudos, observou-se que no TEP a sua elevação sérica ocorria em 51% dos casos e que isso estava diretamente relacionado ao aumento de mortalidade, 16,8% *versus* 1,7% no grupo com valores normais^29^. Por outro lado, valores normais do BNP (< 100 ng/L) praticamente descartam TEP complicado em pacientes de baixo risco clínico (PESIs = 0)^23^. Valores falsos positivos podem aparecer em situações de estimulação neuro-humoral, inflamação e isquemia^30^.

### Disfunção do VD

a) Troponina T cardíaca (TT): é um marcador de lesão miocárdica que no TEP normotenso está associado a maior mortalidade (RR: 5,9); porém, não deve ser usado como parâmetro único. Em revisão que avaliou nove estudos, 28% dos pacientes com TEP normotensos tinham a TT positiva (> 0,1 ng/mL) e a mortalidade nesse grupo foi de 15,9% *versus* 3,4% no grupo sem elevação desta^29^.
b) TT de alta sensibilidade (TTas): parece ser um método com melhor valor preditivo (VP) negativo, pois permite inclusive o ajuste com a idade^29^.
c) Proteína cardíaca de união aos ácidos graxos (*heart-type fatty acid binding protein*, H-FaBP): outro marcador de lesão cardíaca que é liberado no TEP grave, porém não está habitualmente disponível nos exames laboratoriais de rotina^29^.

### Massa trombótica

a) Trombose venosa concomitante: esse marcador de massa trombótica global ajuda na avaliação do risco de recidiva do TEP. No estudo PROSPECT, a presença de trombo no eco-Doppler colorido venoso profundo de MMII é um marcador independente de morte em pacientes normotensos e que não receberam TFS^22^.
b) Dímero D: Valores acima de 2.000 µg/L no registro RIETE se associaram com maior mortalidade (7% *versus* 2,7%)^31^.

Outros marcadores prognósticos no TEP, porém ainda não validados, são a hiponatremia^32^ e o aumento do ácido lático^33^.

## COMBINAÇÃO DE FATORES

Como nenhum dos marcadores tem capacidade suficiente para corroborar a TFS individualmente, podemos utilizar a combinação destes para tentar otimizar a sua indicação. Quando se combina um marcador de lesão com um marcador de dilatação do VD, o VP de complicação ou morte na TFS aumenta entre 15 e 20%, e se a isso se acrescentam os fatores clínicos de risco (por exemplo, PESIs ≥1) ou um marcador de massa trombótica, o VP aumenta em 20 a 30%^22^.

No escore BOVA, em 2.874 pacientes com TEP e normotensos, sobre sete pontos possíveis, ter quatro (grau III) se relacionou com mortalidade de 10,5% ao mês e com complicações do TEP em 29 a 37%. Nesse escore, a PA sistólica abaixo de 100 mmHg, a TT positiva e a dilatação do VD (ecocardiograma ou TC) somavam dois pontos cada e o aumento da FC (> 100 bpm), um ponto^34^.

No estudo PROSPECT, em 591 pacientes com TEP e normotensos, avaliou-se a TT, o ecocardiograma e a ecografia de MMII. Cada um desses parâmetros isolados tinha um VP positivo de mortalidade de aproximadamente 10%; porém, quando se combinavam dois deles, esse valor chegava a até 25% no grupo com PESI de alto risco^22^.

O escore FAST modificado substituiu a H-FaBP pela TTas e avaliou 388 pacientes com TEP e normotensos. Na presença de síncope ou TTas se atribuía 1,5 pontos e na de taquicardia (> 100 bpm), um ponto. Se o paciente atingia três ou mais pontos, era considerado de alto risco^29^.

Existem algoritmos que associam marcadores clínicos com laboratoriais e de imagem, porém ainda não está clara qual a combinação ideal para determinar o paciente com TEP de risco intermediário e mau prognóstico.

### Risco de sangramento com agentes fibrinolíticos sistêmicos

O sangramento maior ou fatal por hemorragia do SNC é a principal limitação para o uso generalizado da TFS no TEP, especialmente no paciente considerado de risco intermediário^35^. Uma revisão sistemática de trabalhos randomizados de TFS no TEP, demostrou uma incidência de 9,24% de sangramento maior em 2.115 pacientes *versus* 1,45% no braço da heparina^12^. No estudo *Pulmonary Embolism Thrombolysis* (PEITHO), observou-se 11,5% de sangramento maior na TFS *versus* 2,5% no uso da heparina. Quando se avaliou apenas o sangramento do SNC, observaram-se taxas de dois e 0,2% respectivamente^36^.

Nos registros como o *International Cooperative Pulmonary Embolism Registry* (ICOPER), que incluem uma amostra mais realista da população geral, é observada uma maior quantidade de sangramentos maiores, que chega a superar os 20%^37^. Em outras séries, observa-se uma frequência de sangramento de SNC superior a 2%, com uma mortalidade de 55%^38^. Esses dados demonstram ser primordial a identificação do paciente que está sob risco maior de sangramento^39^.

Os critérios propostos pela ACCP em 2016^40^ são:

Contraindicação absoluta para o uso de trombolíticos: história de sangramento em SNC, acidente vascular isquêmico (< 3 meses), cirurgia de SNC ou trauma maior (< 1 mês), sangramento ativo, tumor de SNC ou anomalia estrutural cerebral, dissecção aórtica, ressuscitação cardiopulmonar prolongada (> 10 minutos), discrasia sanguínea, INR > 1,7 e plaquetopenia (< 100.000/mm^3^).

Contraindicações relativas para o uso de trombolíticos: PA sistólica > 180 mmHg, idade > 75 anos, sangramento recente (fora do SNC), gestação, pericardite aguda, derrame pericárdico, malformações vasculares, retinopatia diabética, anticoagulação, punções vasculares não compressíveis e recentes, cirurgia recente (< 2-4 semanas) e úlcera péptica ativa.

Fatores de risco de sangramento do SNC com o uso de trombolíticos^39^^,^ 41: idade > 65 anos, peso < 50 kg, anemia, insuficiência renal ou hepática, neoplasia em atividade, diabetes melito, uso de antiagregantes plaquetários ou anti-inflamatórios não hormonais, sexo feminino e risco de sangramento RIETE > 4 (Tabela 1)^42^.

**Tabela 1:** Escala RIETE de risco de sangramento.

**Escala de risco de sangramento RIETE modificada**	
**Variável**	**Pontos**
Hemorragia maior recente (< 1 mês)	2
Creatinina > 1,2 mg/dL	1,5
Anemia	1,5
Câncer	1
Idade > 75 anos	5
Risco baixo: 0; Risco intermediário: 1-4; Risco alto: > 4	

Essa extensa lista de fatores de risco e de contraindicações deixa cerca de 50% dos pacientes de TEP impossibilitados de receber a TFS, porém em algumas situações especiais podemos pensar nessa possibilidade^12^. O sangramento na TFS ainda permanece sob debate, e opções como diminuição da dose em *bolus* preconizada e seu uso local com cateter (trombólise fármaco-mecânica) já são utilizadas e começam a mostrar resultados promissores.

### Dose de fibrinolíticos sistêmicos no TEP

Já está bem estabelecido que a TFS pode ser administrada em até 14 dias após o evento, porém quanto mais cedo isso for feito, preferencialmente antes de 72 h, maior a chance de reperfusão clinicamente relevante. As infusões de 2 h de duração provocam menos sangramentos maiores quando comparadas às de 24-48 h, e devem ser utilizadas preferencialmente. A determinação do local de punção para a angiografia deve ser feita com controle ecográfico e por um médico experiente^3^^,^ 14 ^,^ 16. As duas opções para a TFS no Brasil são: a alteplase e a estreptoquinase.

1. Alteplase (rtPA): ativador tissular de plasminogênio recombinante (Actilyse®, Boehringer Ingelheim), disponível em frascos de 50 mg. Específico para a fibrina, sua vida média é de apenas 5 minutos e é catabolizado no fígado. A dose utilizada no TEP é de 10 mg infundido em *bolus* (1 a 2 minutos) e 90 mg em infusão contínua por 2 h.

No estudo MOPPET avaliou-se a dose de 50% em *bolus* de 15 minutos em 121 pacientes com TEP maciço avaliados pela angiografia (trombo em pelo menos 70% da árvore vascular). Não houve diferença na mortalidade e na recorrência, porém houve no desenvolvimento da HP crônica em 2 anos (57 *versus* 16%)^43^.

1. Estreptoquinase (Streptase®, CSL Behring) em frascos de 1,5 milhões unidades internacionais (UI). A dose utilizada no TEP é a infusão de 1,5 milhões UI em 2 h ou 250.000 UI em 30 minutos e após, 100.000 UI por hora em 12 a 24 h. Trata-se de uma proteína não enzimática isolada do estreptococo *β* hemolítico do grupo A de Lancefield, e pode induzir a formação de anticorpos. Sua vida média é de cerca de 12 minutos e sua inativação é feita pelos anticorpos circulantes produzidos pela exposição prévia ao estreptococo, na ausência deles a sua meia vida pode chegar a até 83 minutos.

Logo após o término da infusão da TFS, deve-se reiniciar a terapia com heparina não fracionada (HNF), caso o tempo de tromboplastina parcial ativada (*activated partial thromboplastin time*, PTTa) esteja menor que duas vezes o valor basal. Se em 24 h o paciente permanecer estável, pode-se trocar a HNF por heparina de baixo peso molecular. Outro parâmetro que se pode utilizar para reiniciar a HNF é o nível sérico de fibrinogênio, que deve estar acima de 100 mg/dL^15^^,^ 16 ^,^ 44.

### Paciente com TEP maciço (alto risco)

O paciente com IH é um paciente crítico e com mortalidade muito alta em poucas horas. Esse paciente se apresenta hipotenso (PA sistólica < 90 mmHg) e com sinais de hipoperfusão periférica (sudorese, déficit sensorial, oligúria, hipotermia, taquicardia e hipoxemia). No estudo ICOPER, o TEP maciço apresentou uma mortalidade de 52,4% ao mês, e se o paciente apresentava choque cardiogênico ou parada cardiorespiratória, esta chegava a até 70%^45^. Nesses casos, o paciente deve ser rapidamente submetido a TFS^46^^,^ 47.

Um parâmetro sensível de gravidade e que auxilia na definição de paciente de alto risco é o *shock index*, que é a simples divisão da FC pela PA sistólica. Qualquer número superior a um denota um marcador de gravidade^48^.

Esses pacientes têm comprometimento de pelo menos 50% do território da artéria pulmonar na angiografia, e esse dado pode-se objetivar com o índice de Miller, que pontua a gravidade do TEP de acordo com o número de ramos obstruídos^49^. A definição de TEP massivo pelas imagens se dá quando se tem mais de 17 pontos de 34 possíveis (índice de Miller > 0,5).

As evidências em que se baseia a indicação da TFS no TEP maciço são muito escassas. O único trabalho randomizado sobre o assunto avaliou apenas oito pacientes com hipotensão e o resultado foi categórico: os quatro pacientes que receberam a TFS com estreptoquinase em 2 h sobreviveram e os quatro tratados com heparina morreram por TEP^49^.

Outros trabalhos da década de 70 incluíram pacientes com TEP grave tratados com TFS, porém usaram critérios angiográficos de gravidade e diferentes esquemas de tratamento^9^^,^ 50 ^-^ 52.

Esse nível de evidência fraco se reflete na diretriz da ACCP de 2012, na qual o uso da TFS no TEP maciço apresenta um nível de evidência 2C^14^.

### Paciente com TEP submaciço (risco moderado)

Esse é o grupo de pacientes mais complexos para se definir a indicação da TFS^52^.

No estudo MAPPET 3, que avaliou 256 pacientes com HP ou falência de VD, o braço em que foi usado rtPA apresentou redução da IH, de 24 para 10%, porém houve elevação de sangramentos maiores, de 0,8 para 3,6%^10^.

O estudo PEITHO avaliou 1.006 pacientes com TEP submaciço (dilatação e disfunção de VD), e apesar de a TFS corrigir a IH de 5 para 1,6%, houve aumento de sangramentos maiores, de 2,4 para 11,5%, e de sangramentos no SNC, de 0,2 para 2%^36^. Em outros registros, a mortalidade aumentou com a TFS quando comparada à heparina^53^.

Outra metanálise que avaliou quatro estudos e 1.775 pacientes com TEP submaciço evidenciou uma queda da mortalidade de 3,9% no braço da heparina e de 2,2% com a TFS, o que se deve ao aumento de sangramento maior de 3,4 para 9,2% com a TFS^12^.

Outras duas metanálises recentes apresentam resultados semelhantes^54^^,^ 55. Entretanto, os únicos dois estudos que avaliaram os pacientes a longo prazo demonstraram diminuição da HP com a TFS^11^^,^ 43. Com base nesses dados descritos, recomenda-se a TFS no TEP submaciço para aqueles pacientes que têm baixo risco de sangramento e evidências de deterioração clínica^21^^,^ 40.

### Alternativas para a TFS

a) Trombolíticos dirigidos por cateter (TDC)

O conceito de combinação de baixas doses locais de fibrinolíticos associados à lise mecânica do trombo vem se estabelecendo com o passar dos anos^56^^,^ 57. Essa associação permite um efeito hemodinâmico imediato, devido à ruptura mecânica do trombo aliada a uma maior concentração do agente fibrinolítico no local, evitando a ação sistêmica da terapia fibrinolítica e diminuindo o risco de sangramentos maiores^58^^-^ 60.

O estudo SEATTLE II com 150 pacientes com TEP maciço ou submaciço tratados com 20 mg de rtPA demonstrou em 30% destes, a correção da disfunção de VD e da HP em 24 h, sem nenhum sangramento de SNC^61^, e o estudo ULTIMA, com 59 pacientes, também observou uma rápida melhora dos parâmetros de dilatação de VD sem sangramentos maiores (apenas 3,6% de sangramentos menores)^62^.

As limitações dessa técnica são a necessidade de uma equipe especializada, o custo de material e ainda uma escassa evidência científica^21^^,^ 40, ficando seu uso restrito aos pacientes que não têm boa evolução com o tratamento sistêmico.

b) Embolectomia cirúrgica

Esse método requer esternotomia e *by-pass* cardiopulmonar, além de uma equipe cirúrgica experiente disponível, e só é recomendado quando há contraindicação para o uso de fibrinolíticos ou naqueles casos refratários à TFS^40^.

O papel dos anticoagulantes orais diretos (DOACs) na TFS

Os DOACs foram excluídos dos estudos de TEP quando se preconizava a TFS, e portanto, até o momento, o uso simultâneo dos mesmos com agentes fibrinolíticos está contraindicado por falta de evidência científica^63^.

## CONCLUSÃO

O uso da TFS, sistêmica ou local, em casos de TEP ainda requer evidência científica mais densa, apesar de muitos trabalhos já demonstrarem melhoras dos parâmetros hemodinâmicos e da sobrevida, especialmente em pacientes com IH. Nos pacientes com TEP e estáveis hemodinamicamente, porém com sobrecarga de VD, há que se levar em consideração o risco de sangramento maior ou no SNC, para avaliar o real benefício da TFS. Portanto, a TFS só deve ser considerada naqueles pacientes com baixo risco de hemorragia e cuja evolução clínica for desfavorável, a despeito da correta anticoagulação. O uso de TDC, se disponível, poderá ser uma opção mais segura, porém necessita de maior evidência científica para ser recomendada de rotina.
