# Supplementary material for: Terapia fibrinolítica sistêmica no tromboembolismo pulmonar
Source: J Vasc Bras. 2017 Apr-Jun;16(2):119–27. [Article in Portuguese] doi: 10.1590/1677-5449.007316 (PMC5915860; doi:10.1590/1677-5449.007316)
Supplement: Translation

# Translation: Systemic fibrinolytic therapy in pulmonary thromboembolism

**Authors:** José Manuel Ceresetto, Marcos Arêas Marques

**Affiliations:** 1 Hospital Britânico de Buenos Aires, Buenos Aires, Argentina.; 2 Universidade do Estado do Rio de Janeiro – UERJ, Hospital Universitário Pedro Ernesto – HUPE, Rio de Janeiro, RJ, Brazil.

**Keywords:** fibrinolytics, pulmonary embolism, hemorrhage

## Abstract

Pulmonary thromboembolism remains a major therapeutic challenge for specialists and, despite investment and the consequent developments in diagnosis, prophylaxis, and treatment, the condition is still the leading cause of avoidable deaths in hospital settings. There is still great uncertainty with relation to the profile of patients who will actually benefit from systemic fibrinolytic treatment, without being exposed to serious risk of bleeding. There are tools that can help to identify patients who will benefit, including risk stratification and estimation of the prognosis of the event, with clinical scores for right ventricular failure, markers of right ventricular dysfunction and dilatation, and thrombotic mass assessment, whether alone or in combination. The only points of consensus with relation to fibrinolytic therapy for treatment of pulmonary thromboembolism are as follows: it should not be routinely indicated, none of the scores or markers alone should be used to justify its use, and patients with hemodynamic instability are the most likely to benefit. Furthermore, each case should be evaluated for risk of bleeding, especially central nervous system bleeding.

## INTRODUCTION

The first published reports of thrombolysin (a mixture of streptokinase and plasminogen) for fibrinolytic treatment of pulmonary thromboembolism (PTE) were based on studies with 43 patients (30 normotensive) undertaken between 1958 and 1964 at the Sloan Kettering Institute in New York and reported results that were neither cause for enthusiasm nor for concern.^1^ To date, the scenario remains similar; there has been no significant improvement in the results published over the years, but prohibitive complications secondary to use of this treatment have not been observed, and the uncertainty related to which patients will benefit most has not been resolved either.

For the last 30 years, there has been double-digit mortality (17%) during the first months after PTE.^2^^-^ 4 Deaths secondary to PTE are the result of acute right ventricular failure (RV), caused by sudden obstruction of a pulmonary artery and culminating in cardiogenic shock due to progressive failure of left ventricle (LV) preload.^5^

Systemic fibrinolytic therapy (SFT) is able, in a short time, to reduce thrombotic mass, correct pulmonary hypertension (PH) secondary to PTE, optimize hemodynamic parameters, and improve oxygenation and capillary flow, with rapid relief from symptoms, and even eliminates remnant thrombi in the pelvis or lower limbs, reducing the risk of relapse of pulmonary embolism.^6^

The only study that has compared mortality from SFT with mortality caused by heparin for treatment of massive PTE with hemodynamic instability (HI) was terminated prematurely because of an elevated mortality rate in the heparin arm, while the mortality rate in the SFT arm was zero.^7^ Since then, patients with PTE and HI are the primary candidates for SFT. However, risk of death, recurrence of PTE, and development of chronic PH are more frequent among patients with acute RV failure (submassive PTE);^8^ in view of this, for many years there has been debate on expanding use of SFT in these patients, since they are much more common than cases with HI.^9^ However, published studies in which use of heparin has been compared with SFT for submassive PTE have not reported significant reductions in mortality with SFT and, because of this, its use has become established as restricted to just the 3 to 5% of patients with PTE who have HI.^8^

Those who advocate using SFT in submassive PTE argue that the populations evaluated in clinical trials are subject to bias, such as, for example, in the Management Strategies and Prognosis of Pulmonary Embolism-3 (MAPPET 3) study, in which only 31% of the patients had RV dilatation, resulting in 2% mortality in the heparin arm of the clinical trial, well below the 10% that would be expected and which, since salvage with SFT was permitted in this arm, favored survival in the control group.^10^ There have also been criticisms of the size of the samples studied, since, summing all patients from randomized studies, only slightly more than 1000 patients have been given SFT, which is considered insufficient.^10^ It should however be noted that these studies also showed significant reduction in secondary parameters, such as rapid improvement of PH and reduction of thrombotic mass and RV dilatation.^11^

Naturally, one of the most significant problems related to use of SFT is the increased number of major bleeding events (10 to 20%), particularly in the central nervous system (CNS), with morbidity, including mortality, of up to 55%, which obviously limits its benefits.^12^^,^ 13

As a result, current consensus statements on management of PTE are clear and objective in stating that the only precise indication for using SFT is in patients with HI.^3^^,^ 14 ^,^ 15 The ninth consensus of the American College of Chest Physicians (ACCP), in 2012, suggested that SFT should only be used in the absence of HI in severe PTE with RV failure and even this recommendation was based on a very weak evidence level (2C) and was subject to case-by-case assessment of risks and benefits.^14^

A recently-published study described experience with use of SFT in 20% of American hospitals. Just 2.1% of more than 90,000 patients diagnosed with PTE from 2008 to 2011 were given SFT, and just 13.2% of the subset of patients who needed vasopressors to maintain arterial blood pressure (BP) were given SFT.^16^^,^ 17

The objective of this article is to discuss the risks and benefits of SFT for treatment of PTE and which patients actually benefit from this treatment. The discussion is based on the results of a bibliographic review of articles listed on PubMed that were published in English during the last 10 years.

## RISK STRATIFICATION IN PTE

To identify which patients are most suitable for SFT, it has been proposed that they should be classified by risk of death and prognosis of the event. Many studies identify secondary markers of PTE severity, referred to as complications of PTE, such as need to repeat SFT, use of ventilatory and hemodynamic support with inotropic agents to maintain BP, cardiopulmonary resuscitation, and relapse of the event.^18^

Pulmonary thromboembolism can be classified as low risk (normotensive, without RV failure), moderate risk (normotensive, with RV failure), or high risk (refractory hypotension).^19^^,^ 20

While practical, this classification involves problems related to the definition of RV failure, whether by dilatation or dysfunction, based on unclear and subjective criteria and linked with an inability to accurately predict its morbidity and mortality.^20^^-^ 22 This reveals a need to evaluate more trustworthy markers of severity to contribute to treatment decisions. In other words, to define whether full anticoagulation with heparin is sufficient or if it is necessary to employ SFT with this profile of patients who may progress to worse prognoses.^6^

This is undoubtedly the major challenge to be overcome in PTE treatment.

### Clinical prognostic criteria

With the exception of patients with systolic BP below 90 mmHg due to cardiogenic shock, assessment of certain clinical parameters such as hepatic congestion, jugular distension, systolic murmur in the pulmonary valve topography and, especially, indirect signs of shock (hypothermia, mental confusion, syncope, tachycardia, progressive hypotension, and hypoxemia demanding respiratory assistance) improves the predictive capacity of complications in PTE.^23^

The best-known clinical risk score is the Pulmonary Embolism Severity Index (PESI), which classifies patients into five risk categories. Patients in lower-risk categories (categories I and II) had mortality of 2% at 1 month, whereas mortality among those in the highest risk group (category V) was 14%.^24^ In 2010, a simplified PESI was published with five easy-to-measure parameters: age > 80 years, active cancer, chronic cardiopulmonary disease, heart rate (HR) > 110 bpm, and systolic BP < 100 mmHg, each worth 1 point. This score has been validated and is useful for identifying patients with good prognosis^25^ and differentiates those with low-risk PTE (PESI = 0), with mortality of 1.1% at 1 month, from those with moderate/high risk (PESI ≥ 1), with mortality of 8.9% at 1 month.

### Right ventricle dilatation

a) Electrocardiogram: although this is a method that is easy to use, it does not figure on the majority of algorithms for PTE assessment. It can reveal abnormalities such as a new right branch blockage, elevation or depression of the anteroseptal ST segment, and/or the S1Q3T3 pattern.
b) Echocardiogram: this is a very useful tool for assessment of RV failure, since it provides a dynamic image of contraction function, can be conducted at the bedside in an unstable patient and is noninvasive, meaning it can be repeated regularly to monitor SFT. Additionally, it allows measurement and evaluation of RV size and its relationship to the LV (RV/LV ratio) and detection of hypokinetic RV free wall, paradoxical movement of the interventricular septum (McConell’s sign), absence of inspiratory collapse of the inferior vena cava, intensity of PH, and tricuspid valve regurgitation.^23^^,^ 26 Limitations include the facts that it is an examiner-dependent technique and that to date there are no standardized criteria for definition of RV failure.
c) Computed tomography (CT): this is a very useful technique for assessment of RV diameter and its ratio to the LV (if greater than 0.9 at the level of the tricuspid and mitral valves it characterizes a dilated RV). Data from the Prospective Investigation of Pulmonary Embolism Diagnosis II trial (PIOPED II) showed that this parameter was linked with increased mortality (RR: 1.27),^27^ and a meta-analysis observed that it increased mortality 2.5 times.^28^ It has limited value as an exclusive method and tends to amplify the number of patients with a dilated RV (in PIOPED II, 50% of normotensive patients had a dilated RV).
d) Natriuretic peptide type B (BNP) and its terminal fragment NT-pro-BNP: these biomarkers are secreted by the myocardium when exposed to excessive dilatation of its fibers, providing an indirect marker of RV dilatation. A review of 13 studies observed that serum levels were elevated in 51% of PTE cases and that this was directly related to increased mortality, 16.8% vs. 1.7% in a subset with normal levels.^29^ Conversely, normal BNP levels (< 100 ng/L) practically ruled out PTE in patients at low clinical risk (PESI = 0).^23^ False positive results can occur in situations with neurohumoral stimulation, inflammation, and ischemia.^30^

### RV Dysfunction

a) Cardiac troponin T (TT): this is a marker of myocardial injury which in normotensive PTE is associated with greater mortality (RR: 5.9), but it should not be used as the only parameter. A review that analyzed nine studies found that 28% of patients with normotensive PTE were TT positive (> 0.1 ng/mL) and mortality in this subset was 15.9% compared with 3.4% in the negative subset.^29^
b) High-sensitivity TT (TThs): this appears to be a method with a better negative predictive value (PV) and enables correction for age.^29^
c) Heart-type fatty acid binding protein (H-FaBP): another marker of cardiac injury that is released in severe PTE, but which is not generally available in routine laboratory tests.^29^

### Thrombotic mass

a) Concomitant venous thrombosis: this marker of overall thrombotic mass is useful for assessment of risk of PTE relapse. In the PROSPECT study, presence of a thrombus on color Doppler ultrasonography of the deep venous system of the lower limbs was an independent marker of death among normotensive patients who did not receive SFT.^22^
b) D Dimer: In RIETE registry data, values over 2,000 µg/L were associated with higher mortality (7% vs. 2.7%).^31^

Other prognostic markers in PTE are hyponatremia^32^ and elevated lactic acid,^33^ although these have not yet been validated.

## COMBINATIONS OF FACTORS

Since none of these markers in isolation has the capacity to endorse SFT, we can employ a combination of them to attempt to optimize treatment decisions. When a marker of RV injury is combined with a marker of RV dilatation, the PV for SFT complications or death increases by 15 to 20%, and if these are combined with clinical risk factors (for example, PESI ≥1) or a marker of thrombotic mass, the PV increases by 20 to 30%.^22^

A study with 2,874 normotensive patients with PTE was used to derive the BOVA scale, on which a score of four out of seven possible points (stage III) is linked with mortality of 10.5% at 1 month and with PTE complications in 29 to 37%. On this scale, systolic BP below 100 mmHg, positive TT, and RV dilatation (on echocardiogram or CT) each score two points and elevated HR (> 100 bpm) scores one point.^34^

The PROSPECT study, with 591 normotensive patients with PTE, investigated TT, echocardiogram, and echography of the lower limbs. Each of these parameters in isolation had a positive PV for mortality of approximately 10%, but when any two of them were combined, the predictive value reached 25% for the group with high risk PESI.^22^

The modified FAST score substitutes H-FaBP with TThs and was investigated with 388 normotensive patients with PTE. In the presence of syncope or TThs, 1.5 points are allocated and tachycardia (> 100 bpm) scores a further 1 point. Patients with three or more points are considered high risk.^29^

There are algorithms that combine clinical markers with laboratory and imaging findings, but it is not yet clear which combination is ideal for identifying intermediate risk PTE patients who have poor prognosis.

### Risk of bleeding with systemic fibrinolytic agents

Major or fatal bleeding with CNS hemorrhage is the primary barrier to generalized use of SFT for PTE, especially in patients considered intermediate risk.^35^ A systematic review of randomized studies of SFT for PTE demonstrated an incidence of 9.24% of major bleeding in 2,115 patients vs. 1.45% in the heparin arm.^12^ The Pulmonary Embolism Thrombolysis study (PEITHO) observed 11.5% major bleeding with SFT vs. 2.5% with heparin. When compared on the basis of CNS bleeding, the rates were 2 and 0.2% respectively.^36^

Data from the International Cooperative Pulmonary Embolism Registry (ICOPER), which includes a more realistic sample of the general population, reveal a higher rate of major bleeding events, exceeding 20%.^37^ In other series, CNS bleeding rates are over 2% and mortality is 55%.^38^ These illustrate the extreme importance of identifying patients at increased risk of bleeding.^39^

In 2016, the ACCP proposed the following criteria:^40^

Absolute contraindications for thrombolytics: history of CNS bleeding, ischemic stroke (< 3 months), CNS surgery or major trauma (< 1 month), active bleeding, CNS tumor or structural cerebral anomaly, aortic dissection, prolonged cardiopulmonary resuscitation (> 10 minutes), blood disorder, INR > 1.7, or thrombocytopenia (< 100,000/mm^3^).

Relative contraindications for thrombolytics: systolic BP > 180 mmHg, age > 75 years, recent bleeding (other than CNS), pregnancy, acute pericarditis, pericardial hemorrhage, vascular malformations, diabetic retinopathy, anticoagulation, uncompressible and recent vascular punctures, recent surgery (< 2-4 weeks), and active peptic ulcer.

Risk factors for CNS bleeding with thrombolytics:^39^^,^ 41 age > 65 years, weight < 50 kg, anemia, liver or kidney failure, active neoplasm, diabetes mellitus, platelet antiaggregants, or nonsteroidal anti-inflammatories, female sex, and RIETE bleeding risk > 4 (Table 1).^42^

**Table 1:** RIETE risk of bleeding scale.

**Modified RIETE risk of bleeding scale**	
**Variable**	**Points**
Recent major hemorrhage (< 1 month)	2
Creatinine > 1.2 mg/dL	1.5
Anemia	1.5
Cancer	1
Age > 75 years	5
Low risk: 0; Intermediate risk: 1-4; High risk: > 4	

This extensive list of risk factors and contraindications excludes 50% of PTE patients from SFT, although in certain special cases its use can still be considered.^12^ Bleeding while on SFT is still a subject of debate, and options such as reducing the recommended dose in bolus and local administration with catheters (pharmaco-mechanical thrombolysis) are already being used and have begun to exhibit promising results.

### Systemic fibrinolysis doses in PTE

It is well-established that SFT can be administered up to 14 days after the event, but the earlier that it is accomplished, preferably within 72 h, the better the chances of achieving clinically relevant reperfusion. Infusions of 2 h duration provoke fewer major bleeding events than those lasting 24-48 h and should be preferred. Puncture sites for angiography should be chosen with ultrasound control and by an experienced physician.^3^^,^ 14 ^,^ 16 The two options available for SFT in Brazil are: alteplase and streptokinase.

1. Alteplase (rtPA): recombinant tissue plasminogen activator (Actilyse®, Boehringer Ingelheim), available in 50 mg vials. Specific to fibrin, its half-life is just 5 minutes and it is catabolized in the liver. The dose employed in PTE is 10 mg infused in bolus (1 to 2 minutes) and 90 mg in continuous infusion over 2 h.

The MOPPET study assessed a 50% dose in a 15-minute bolus in 121 patients with massive PTE assessed by angiography (thrombus in at least 70% of the vascular tree). There was no difference in mortality or recurrence, but there was a difference in development of chronic PH at 2 years (57 vs. 16%).^43^

1. Streptokinase (Streptase®, CSL Behring) in vials containing 1.5 million international units (IU). The dose employed for PTE is infusion of 1.5 million IU in 2 h or 250,000 IU in 30 minutes and then 100,000 IU per hour over 12 to 24 h. This is a non-enzymatic protein isolated from Lancefield group A *β* hemolytic streptococcus, and it can induce formation of antibodies. Its half life is around 12 minutes and it is deactivated by circulating antibodies produced by prior exposure to streptococcus, although in their absence half-life can be as long as 83 minutes.

Soon after infusion of SFT is complete, treatment with unfractionated heparin should be resumed if activated partial thromboplastin time is less than twice baseline. If the patient remains stable for 24 h, unfractionated heparin can be changed for low molecular weight heparin. Another parameter that can be used for resuming unfractionated heparin is serum fibrinogen concentration, which should be above 100 mg/dL.^15^^,^ 16 ^,^ 44

### Patients with massive PTE (high risk)

Patients with HI are critical and have very high mortality in just a few hours. They are hypotensive (systolic BP < 90 mmHg) and have signs of peripheral hypoperfusion (diaphoresis, sensory deficit, oliguria, hypothermia, tachycardia, and hypoxemia). In the ICOPER study, massive PTE was associated with 52.4% mortality at 1 month, and if the patient had cardiogenic shock or cardiorespiratory arrest, it reached 70%.^45^ In these cases, patients should rapidly be put on SFT.^46^^,^ 47

The shock index is a sensitive parameter of severity that is useful for defining patients as high risk and is derived by simply dividing HR by systolic BP. Any number greater than 1 is a marker of severity.^48^

These patients have at least 50% of the territory of the pulmonary artery compromised on angiography and this finding can be made more objective by calculating the Miller index, which scores the severity of PTE according to the number of branches obstructed.^49^ Massive PTE is defined on the basis of images when more than 17 out of 34 possible points are scored (Miller index > 0.5).

The evidence on which indication of SFT for massive PTE is based is very scant. The only randomized trial of the subject only assessed eight patients with hypotension, but the result was categorical: all four patients given SFT with streptokinase for 2 h survived and all four treated with heparin died from PTE.^49^

Other studies in the 1970s included patients with severe PTE who were treated with SFT, but they employed angiographic criteria for severity and different treatment regimens.^9^^,^ 50 ^-^ 52

This weak evidence level is reflected in the 2012 ACCP guideline in which use of SFT in massive PTE has an evidence level of 2C.^14^

### Patients with submassive PTE (moderate risk)

This is the group of patients in which the decision on SFT is most complex.^52^

In the MAPPET 3 study, which assessed 256 patients with PH or RV failure, a reduction in HI from 24 to 10% was observed in the arm in which rtPA was used, but there was an increase in major bleeding from 0.8 to 3.6%.^10^

The PEITHO study assessed 1,006 patients with submassive PTE (RV dilatation and dysfunction) and although SFT corrected HI from 5 to 1.6%, there was an increase in major bleeding, from 2.4 to 11.5%, and in CNS bleeding from 0.2 to 2%.^36^ In other registries, mortality increased with SFT when compared with heparin.^53^

Another meta-analysis evaluated four studies and 1,775 patients with submassive PTE, observing a 3.9% reduction in mortality in the heparin arm and a 2.2% reduction with SFT, which was due to an increase in major bleeding from 3.4 to 9.2% with SFT.^12^

Two other recent metanalyses reported similar results.^54^^,^ 55 However, the only two studies that assessed patients over the long term demonstrated that PH reduced with SFT.^11^^,^ 43 Based on the data described, SFT is recommended in submassive PTE for patients at low risk of bleeding who show signs of clinical deterioration.^21^^,^ 40

### Alternatives to SFT

a) Catheter-directed thrombolysis

The concept of low localized doses of fibrinolytics combined with mechanical lysis of the thrombus has been gaining ground over the years.^56^^,^ 57 This combination offers an immediate hemodynamic effect, because the thrombus is ruptured mechanically, combined with a higher concentration of the fibrinolytic agent at the site, avoiding the systemic action of fibrinolytic therapy and reducing the risk of major bleeding.^58^^-^ 60

The SEATTLE II study of 150 patients with massive or submassive PTE treated with 20 mg of rtPA demonstrated correction of RV dysfunction and of PH within 24 h in 30% of them, with no CNS bleeding,^61^ and the ULTIMA study, with 59 patients, also observed rapid improvement in parameters of RV dilatation without major bleeding (just 3.6% minor bleeding).^62^

The limitations of this technique are the need for a specialized team, the cost of the material, and as-yet scant scientific evidence,^21^^,^ 40 which restricts its use to patients who will not respond well to systemic treatment.

b) Surgical embolectomy

This method demands sternotomy, cardiopulmonary bypass, and availability of an experienced surgical team and is only recommended when there are contraindications to fibrinolytics or in cases refractory to SFT.^40^

### The role of direct oral anticoagulants in SFT

Direct oral anticoagulants were ruled out in studies of PTE where SFT was considered, and so, to date, simultaneous use with fibrinolytic agents is contraindicated because of lack of scientific evidence.^63^

## CONCLUSIONS

Use of SFT for PTE cases, whether systemic or local, still requires more definitive scientific evidence, even though many studies have already demonstrated improved hemodynamic parameters and survival, especially in patients with HI. In patients with PTE who are hemodynamically stable, but have RV overload, the risk of major or CNS bleeding must be considered to assess the true benefits of SFT. Therefore, SFT should only be considered in patients at low risk of hemorrhage and whose clinical progress is unsatisfactory despite correct anticoagulation. Use of catheter-directed thrombolysis, where available, may be a safer option, but stronger scientific evidence is needed before it can be recommended as a routine approach.
